# Methotrexate: who would have predicted its importance in rheumatoid arthritis?

**DOI:** 10.1186/s13075-018-1599-7

**Published:** 2018-05-30

**Authors:** Michael E. Weinblatt

**Affiliations:** Brigham and Women’s Hospital, 75 Francis Street, Boston, MA 02115 USA

Forty years ago, the US Food and Drug Administration approved low dose methotrexate (MTX) for the treatment of active rheumatoid arthritis (RA). MTX was previously approved for a variety of malignancies at high doses and at low doses for psoriasis. Little did one anticipate in 1988 that MTX would change the course of RA. I had the pleasure of being directly involved in the development of MTX in RA and it has been extremely gratifying to see the impact of MTX in RA.

For rheumatologists who have recently completed training, one could not imagine the clinical picture 40 years ago in which many of our patients were disabled, unable to work, in wheel chairs, and had significant pain and deformity. That was the landscape of RA in the 1980s. During my fellowship training (1978 to 1980) the pyramid approach to treatment was used, which included high dose aspirin, the newer non-steroidal anti-inflammatory drug such as ibuprofen, corticosteroids, and slow-acting anti-rheumatic therapy, i.e., gold salts, hydroxychloroquine, d-penicillamine, and immuno-suppressive drugs, including azathioprine and cyclophosphamide. Treatment regimens also included joint injections, short-term hospitalization, physical therapy, and joint replacement surgery. The therapeutic landscape was bleak with failures using total nodal radiation and plasmapheresis. New drug development for RA was essentially non-existent with the only drug under development being oral gold (auranofin), which proved to have limited efficacy and gastrointestinal intolerance.

MTX was not specifically developed for RA. Aminopterin, the parent compound of MTX, was the first successful anti-metabolite used in the treatment of childhood leukemia. In 1951, Gubner and colleagues reported a positive open study using aminopterin in RA and psoriasis [1]. The dermatology community began using low dose weekly MTX for the treatment of psoriasis but hepatotoxicity with long-term treatment was observed. The rheumatology community was not excited about using cancer drugs for such a “benign” disease like RA. There was also greater enthusiasm for using corticosteroids for RA—the Nobel Prize was awarded in 1950 to Hench and colleagues for their pioneering work in this area.

Great credit for MTX development in RA needs to go to a few pioneering community-based rheumatologists, including Rex Hoffmeister in Spokane, Washington and Robert Willkens in Seattle, who initially reported their personal experience with low dose MTX for RA [2, 3]. Despite their initial observations, however, definitive randomized controlled trials were not performed at that time.

Upon completion of my fellowship in 1980, I became interested in studying MTX as a treatment for RA. I was influenced by the reports from Hoffmeister and Willkens and my own experience with MTX in psoriatic arthritis. In 1982, I submitted a 35-patient double-blind crossover study to Lederle Laboratories, the manufacturer of MTX, for support of this study. Initially they responded that “we are not interested in supporting studies of MTX in rheumatoid arthritis but would keep the protocol on file.” Approximately 6 months later I was called by the clinical development team at Lederle to attend a meeting in Pearl River, New York to discuss MTX in RA. Attending the meeting were several other rheumatologists who also had submitted protocols, including Jim Williams and John Ward, Directors of the Cooperative Systematic Studies of Rheumatic Disease program, who proposed a randomized parallel control trial, and Joel Kremer from Albany Medical College, who proposed a long-term study to examine the effects of MTX on the liver. In attendance at that meeting was Harriet Kiltie, MD, the medical director at Lederle Laboratories. She was enthusiastic about supporting research on MTX even though it was now a generic drug. Until recently, I was unaware that Dr. Kiltie had begun her research career at Lederle studying folic acid. The history of research on folic acid and the development of aminopterin and its subsequent use by Sidney Farber in childhood leukemia is beyond the scope of this paper but is of significant historical interest.

Our 35-patient study [4] and the CSSRD trial [5] were the two studies reviewed by the US Food and Drug Administration. The FDA Arthritis Advisory Committee overwhelmingly voted in favor of approval of low dose weekly MTX for RA.

Following the initial placebo controlled trials, comparative trials were performed studying MTX versus azathioprine [6] and auranofin (oral gold) [7]. Long-term prospective studies were also performed, with patients in our initial randomized study being followed over 11 years [8]. Combination studies were then conducted combining MTX with all traditional small molecules, including anti-malarials, gold salts, sulfasalazine, oral gold, azathioprine, and cyclosporine. In addition, early biologic response modifiers, including anti-CD4 therapy, were added to a background of MTX.

Multiple studies were done to characterize and minimize the toxicity profile of MTX. Graciela Alarcon and Sarah Morgan performed important studies of the impact of folic acid in reducing MTX toxicity [9]. I collaborated with Joel Kremer and Graciela Alarcon and others in studies of the effects of MTX on liver and lung [10, 11]. Roff Rau in Germany performed some of the initial radiographic studies that showed slowing of radiographic progression with MTX [12] and was a champion of MTX use in Europe.

The development of MTX was an international effort combining the experience of community rheumatologists, clinical researchers in academic centers, and the clinical research team at Lederle Laboratories. There was great comradery amongst the MTX researchers worldwide with the goal of developing a new effective therapy for RA. MTX has now become the standard of care in RA therapeutics and is a major advance in the treatment of this disease.

## Abbreviations

MTX: Methotrexate
RA: Rheumatoid arthritis
